# Case Report: Peritoneal disease from adrenal cortical carcinoma with hepatic metastases managed with cytoreductive surgery and multiple HIPEC sessions, resulting in survival beyond 13 years

**DOI:** 10.3389/fsurg.2026.1725272

**Published:** 2026-04-01

**Authors:** Angel Camarasa Perez, Ruwanthi Wijayawardana, Nima Ahmadi, David Lawson Morris

**Affiliations:** 1 Nuestra Señora de la Candelaria University Hospital (HUNSC), Santa Cruz de Tenerife, Spain; 2 Liver and Peritonectomy Department, St George Hospital, Sydney, NSW, Australia

**Keywords:** adrenocortical carcinoma, cisplatin, cytoreductive surgery, hyperthermic intraperitoneal chemotherapy, peritonectomy

## Abstract

Adrenocortical carcinoma (ACC) is a rare and highly aggressive malignancy with limited therapeutic options and poor long-term survival, particularly in advanced stages. Complete surgical resection remains the cornerstone of treatment, although recurrence is common and management of metastatic disease is challenging. Cytoreductive surgery combined with hyperthermic intraperitoneal chemotherapy (HIPEC) has shown survival benefits in other intraperitoneal malignancies, but its role in ACC remains uncertain due to the rarity of the disease and the scarcity of evidence. We present the case of a 46-year-old woman diagnosed with left-sided ACC in 2012, initially treated with laparoscopic adrenalectomy and adjuvant radiotherapy. Over the following years, she developed multiple peritoneal and hepatic recurrences, managed with repeated cytoreductive surgeries and systemic therapies, including mitotane-based chemotherapy. From 2014 to 2025, the patient underwent four cytoreductive surgeries combined with HIPEC using different intraperitoneal chemotherapy agents, including cisplatin, doxorubicin, and mitomycin C, each achieving complete cytoreduction. The intervals between procedures reached up to three years of disease-free survival, and the patient tolerated all treatments with minimal morbidity. Thirteen years after the initial diagnosis, she remains alive and functionally well following her most recent cytoreductive surgery and HIPEC for bladder and ileocecal recurrence. This case highlights the potential role of iterative cytoreductive surgery combined with HIPEC in achieving long-term survival and disease control in selected patients with recurrent or metastatic ACC. Although limited evidence precludes definitive conclusions, our findings suggest that the integration of HIPEC with surgical management may represent a valuable therapeutic option warranting further investigation in this rare and aggressive malignancy.

## Introduction

Adrenal cortical carcinoma ACC is a rare condition, and its management presents a significant challenge. Survival in advanced stages does not exceed 15% at 5 years (1), according to the literature. It is known that surgical resection is crucial for the initial management; however, recurrence is the norm (over 70%–80% of cases) (1, 2), and its management becomes more complex, typically involving systemic therapy (3).

On the other hand, it is well known that cytoreductive surgery combined with HIPEC (Hyperthermic Intraperitoneal Chemotherapy) has shown benefits in terms of increased survival in several intraperitoneal carcinomas (primarily ovarian, colon, appendix, and stomach cancers) (4–6). However, its benefit in the management of adrenal carcinoma is unclear, with very limited literature on the subject. Prospective and randomized studies are difficult to conduct in this type of pathology due to its low frequency, challenging management, and the scarcity of high-volume centers.

In this article, we describe the management of a patient with peritoneal disease secondary to adrenocortical carcinoma. She had her first surgery in 2012 and is still alive today, having undergone multiple treatments with cytoreductive surgery and HIPEC using different regimens (Table 1).

**Table 1:** Intraperitoneal treatments summary.

Treatment type	Year	HIPEC
Surgery (adrenalectomy)	2012	-
Surgery (liver and peritonectomy)	2014	Cisplatin (100 mg) 58 mg/m^2^ at 41.5°C for 60 min.
Surgery (peritonectomy)	2019	Doxorubicin (26 mg) 14 mg/m^2^ and mitomycin C (22 mg) 12 mg/m^2^ at 41.5°C for 90 min
Surgery (peritonectomy)	2022	Cisplatin (130 mg) 72 mg/m^2^ at 41.5°C for 60 min.
Surgery (peritonectomy)	2025	Cisplatin (130 mg) 80mg/m^2^ at 41.5°C for 60 min

## Materials and methods/case report

A 46-year-old woman with a suspected left adrenal gland tumor, and no other relevant medical history, underwent laparoscopic adrenalectomy in 2012 at Tasmanian Hospital (Australia). The specimen was opened intraperitoneally, and fragmented removal was performed. The pathological diagnosis was adrenal cortical carcinoma, with no metastasis at the time of diagnosis. She received adjuvant treatment with abdominal radiotherapy.

Ten months after the initial surgery, she presented with recurrence, showing 7 peritoneal nodules on follow-up computed tomography (CT). A new surgical intervention was performed, involving resection and diathermy of these nodules.

Six months later, the patient presented with new pathological peritoneal nodules on CT, along with hepatic metastases. Systemic chemotherapy was initiated, consisting of mitotane, doxorubicin, cisplatin, and etoposide. She was later maintained on monotherapy with mitotane until completing a year of treatment.

After a good response in the hepatic metastases but persistent peritoneal nodules, she was referred to the Peritonectomy Unit at St George Hospital (Sydney, Australia). In August 2014, she underwent cytoreductive surgery combined with HIPEC, with a peritoneal carcinomatosis index (PCI) of 5. Procedures included omentectomy, splenectomy, left salpingectomy, left oophorectomy, and resection of a perinephric mass, resulting in a CC0 cytoreductive surgery and HIPEC with **cisplatin (100 mg) 58 mg/m^2^ at 41.5 °C for 60 min.**

She was free of disease until 2017, when two hepatic metastases (no peritoneal disease) were diagnosed in segments 4 and 8, which were surgically resected.

In 2019 (5 years from previous HIPEC), a new peritoneal recurrence occurred, requiring further surgery. A hysterectomy, pelvic peritonectomy, and bowel resection with anastomosis were performed, combined with HIPEC using **doxorubicin (26 mg) 14 mg/m^2^ and mitomycin C (22 mg) 12 mg/m^2^ at 41.5 °C for 90 min**. The pathological result was positive for adrenocortical carcinoma. After recovery, she received systemic chemotherapy with doxorubicin, etoposide, and carboplatin. After two cycles of doxorubicin, the treatment was switched to mitotane. Systemic therapy was completed after 5 months.

In 2021, a new hepatic relapse was detected with a lesion in segments 2–3. Systemic treatment with carboplatin and etoposide was administered, and subsequent surgery involving bisegmentectomy of segments II-III, with no evident peritoneal disease.

At the end of 2022, a new left diaphragmatic nodule was observed. Surgery was decided, revealing a left retrogastric mass with infiltration of the left diaphragmatic dome and tail of the pancreas, as well as multiple small and diffuse nodules. Resection of the left diaphragmatic dome, distal pancreatectomy and subtotal gastrectomy with Roux-en-Y reconstruction were performed. HIPEC were administered with **cisplatin (130 mg) 72 mg/m^2^ at 41.5 °C for 60 min.**

Pathological result compatible with metastasis of ACC and free margins.

Complete remission until the beginning of 2025, when a control image shows a new nodule in the bladder causing ureteral dilation. New cytoreductive surgery with HIPEC was proposed. A significant implant was observed at the bladder level, without involvement of the trigone, as well as disease at the ileocecal level. Ileocecal resection and partial bladder resection were performed, without ureteral involvement. HIPEC therapy with **cisplatin (130 mg) 80 mg/m^2^ at 41.5 °C for 60 min**. PCI of 5 and CC0 resection. Pathological result of ACC in bladder nodule.

The patient was discharged 9 days after surgery, without incident.

## Discussion

While the benefits of intraperitoneal chemotherapy for various intra-abdominal cancers are well-established, there is no clear evidence regarding its use in advanced-stage ACC with peritoneal disease. What is clarified in this type of tumor is that a complete oncological resection is crucial for the prognosis.

A review of the literature reports HIPEC therapies with cisplatin, doxorubicin, and melphalan, with no clear differences regarding the benefit of one over the other. This can be attributed to factors such as the low prevalence of the disease and the evolving nature of this therapy and its indications, which have not yet clarified the best approach for improving survival.

All intraperitoneal chemotherapy regimens described in the literature were used in this patient, with favorable outcomes. Additionally, mitomycin C combined with doxorubicin was used with good results, providing a 3-year disease-free survival in our patient (Table 1). Treatment with mitomicyn C could be considered in the future as a possibility in this type of cancer since It has not been described previously in the literature and had a similar response to cisplatin (3 years).

Our case involves a young patient who has undergone both surgical therapy and combined HIPEC therapy as many times as needed. A total of 8 surgeries were performed, 4 of them were cytoreductive surgery with HIPEC. The treatment used in this case has reported a 13 years survival, at least up to this point. This increased survival, compared to the average for similarly advanced stages, can be attributed to the multiple cytoreductive surgeries combined with HIPEC. This is probably the most relevant aspect in this case, what has not been previously described, with the application of as surgeries as were needed, and the complete resection of the disease. Notably, she has had minimal morbidity and no need for reintervention.

## Conclusion

Cytoreductive surgery may be a good approach in the management of advanced ACC. The addition of HIPEC can significantly slow the progression of the disease, in conjunction with systemic therapy. Although there is limited literature to support this, our patient has had a favorable outcome, with survival far exceeding the average.

However, this is a unique case, not previously described in such a manner, whose management has allowed her survival. It does not provide definitive evidence, but it opens up a treatment field that requires studies to provide more solid evidence for its applicability.

## Data Availability

The original contributions presented in the study are included in the article/Supplementary Material, further inquiries can be directed to the corresponding author.
